# Dermoscopy of early melanomas: variation according to the anatomic site

**DOI:** 10.1007/s00403-021-02226-x

**Published:** 2021-03-26

**Authors:** Linda Tognetti, Alessandra Cartocci, Elisa Cinotti, Elvira Moscarella, Francesca Farnetani, Cristina Carrera, Aimilios Lallas, Danica Tiodorovic, Caterina Longo, Susana Puig, Jean Luc Perrot, Giuseppe Argenziano, Giovanni Pellacani, Gennaro Cataldo, Alberto Balistreri, Gabriele Cevenini, Pietro Rubegni

**Affiliations:** 1 Dermatology Unit, Department of Medical, Surgical and Neurosciences, University of Siena, Siena, Italy; 2 Bioengineering and Biomedical Data Science Lab, Department of Medical Biotechnologies, University of Siena, Siena, Italy; 3 Dermatology Unit, University of Campania Luigi Vanvitelli, Naples, Italy; 4 Department of Dermatology, University of Modena and Reggio Emilia, Modena, Italy; 5 Melanoma Unit, Department of Dermatology, University of Barcelona, Barcelona, Spain; 6 First Department of Dermatology, Aristotele University, Thessaloniki, Greece; 7 Dermatology Clinic, Medical Faculty, Nis University, Nis, Serbia; 8 Centro Oncologico ad Alta Tecnologia Diagnostica, Azienda Unità Sanitaria Locale-IRCCS di Reggio Emilia, Reggio Emilia, Italy; 9 Dermatology Unit, University Hospital of St-Etienne, Saint Etienne, France

**Keywords:** Dermoscopy, Melanoma, Body site, Early diagnosis, Dermoscopic features

## Abstract

To date, is yet to be elucidated whether the body location of cutaneous melanoma can significantly affect an early dermoscopic diagnosis and, consequently, if it can be regarded as a prognostic factor. To investigate the dermoscopic appearance of early melanomas (EMs) at different body sites; to test the ability of dermoscopists in recognizing specific dermoscopic features in EMs. A pool of 106 experienced dermoscopists evaluated the presence of 10 dermoscopic features assumed as suggestive of malignancy among 268 images of EMs with ambiguous appearance located at 16 body sites. According to 720 evaluations, EMs of the “upper extremities” showed a prevalence of early atypical lentiginous features. EMs of the “anterior trunk” exhibited the lower rate of recognition for all features. EMs of the “rear trunk” can be regarded as an intermediate area, showing high recognition rates of regression-related and chronic-traumatism-related features.

## Introduction

Cutaneous melanoma (MM) incidence has been rising in the last decades, and, in parallel, the attention for its etiology and predisposing factors [2, 3, 5, 7–12]. In this context, the body distribution was of interest both for the clinical relevance and for a better understanding of the etiology of the tumor itself [17–19, 24, 27, 30]. While similar incidence rates for usually and intermittently exposed body sites were reported in the 80s [5, 8, 11, 12, 17], an age-related trend appeared in the last decades, due to changes in clothing and photo-exposure habits: under the age of 50, the upper back showed the highest incidence rates in both sexes [2, 3], followed by the lower limb in women used to intermittent sun exposure [10, 30]; over the age of 50, the head was mostly involved [7, 24].

The diagnostic utility of dermoscopy for the management of melanocytic skin lesions is today undoubted [13, 14, 26]. In particular, the recognition of a series of features generally accepted as suggestive of malignancy is well established and recommended to recognize MM [27], being the number of displayed malignancy-related features dependent from the MM stage. To date, some specific dermoscopic clues are well documented for facial and palmo-plantar MM and demonstrated to be useful in early diagnosis of early melanomas (EMs) (i.e., stage 0 and I) [6, 21, 23]. However, it remains to be described if and/or which specific features are exhibited by EMs at different body sites excluding palms, soles and face. Moreover, it is yet to be elucidated if and how the body location of an EM affects its dermoscopic appearance and, consequently, its impact on the recognition by dermoscopists [22, 25, 29].

In this study, we first aimed to describe, in a large dataset of EMs, developed at different body sites of the trunk and extremities, the distribution of ten dermoscopic features currently accepted as suggestive for malignancy among [29]; second, to test if dermoscopists were adequately trained to recognize specific subsets of dermoscopic features in EMs localized at four different macro-areas (anterior/posterior trunk, upper/lower extremities) and, third, to individuate at which body site EM can be considered featureless.

## Methods

### Study design and population

This retrospective study was approved by local ethical committee (Protocol No. 16801); all data were deidentified before use. A total of 268 cases of EMs including in situ, stage I, IIA (pt3a) [20], were retrospectively collected from the whole body surface: lesions were consecutively excised during skin tumor screening activity of dermatologic units from 8 different European centers [29]. Only lesions that were judged as “challenging” according to three out four expert dermoscopists were selected, namely: those cases that, based on the dermoscopic appearance only, may not be clearly differentiated from a dysplastic nevus; thus, being the dermoscopic diagnosis of malignancy was not obvious, they could be considered dermoscopically ambiguous cases. Facial, palmo-plantar and mucosal sites were excluded due to peculiar dermoscopic patterns. Patients, 146 males (54.5%) and 122 females (45.5%), had a mean age of 56 ± 16 years. One dermoscopic, polarized, > 1.5MPx image per case was collected.

### Dermoscopic analysis

**Fig. 1:**
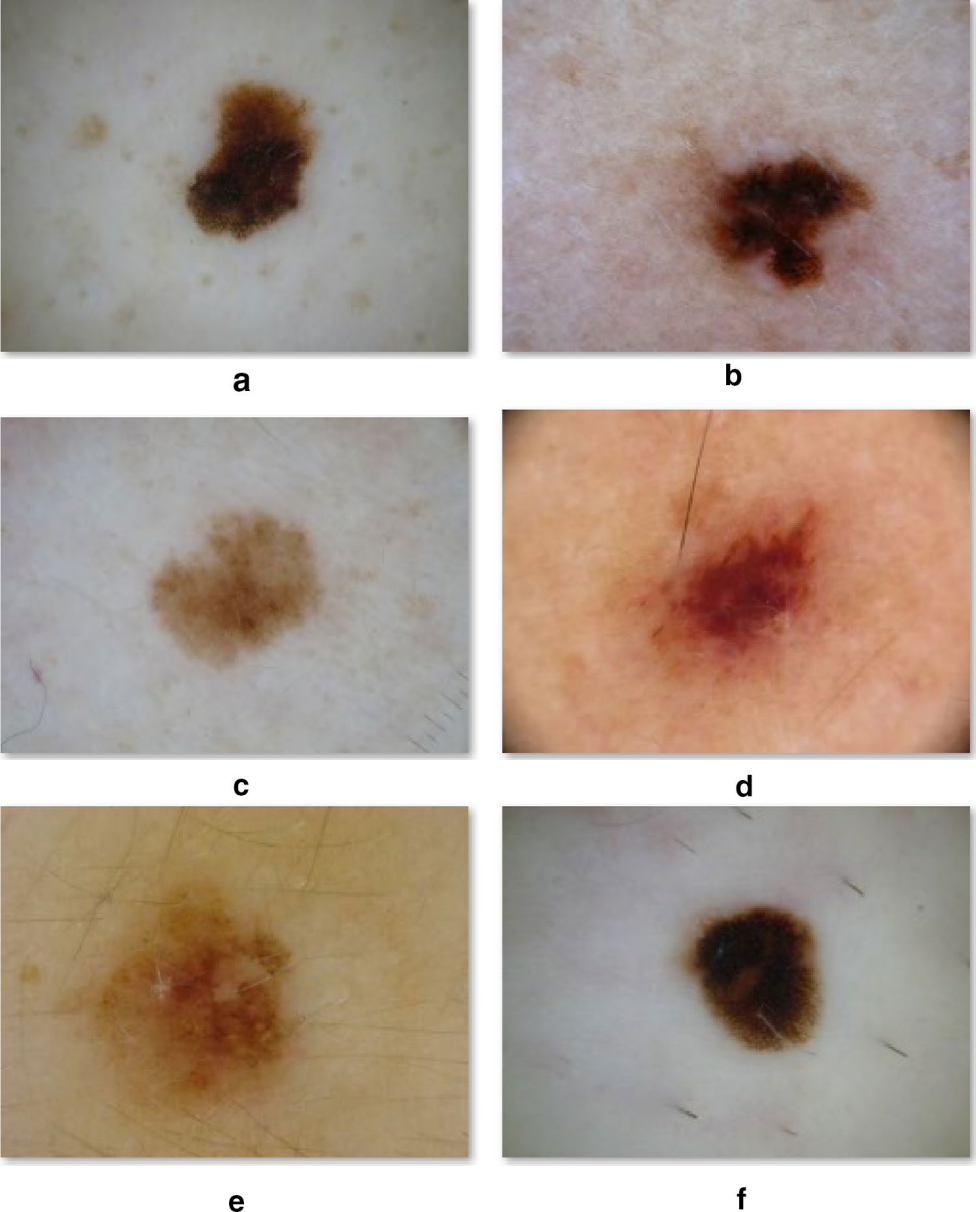
Examples of early melanomas (EM) of 6 mm maximum diameter at different anatomical sites, including the arm of a 39-year-old male (**a**), the upper back of a 34-year-old female (**b**), the breast of a 44-year-old female (**c**), the chest of a 48-year-old male (**d**), the abdomen of a 23-year-old female (**e**) and the leg of a 26-year-old male (**f**). The dermoscopic feature “atypical network” largely varies among different body sites, e.g.: dark and pronounced in EM of the posterior trunk and extremities, light and delicate in EM of the anterior lower trunk

All images were independently evaluated in a teledermoscopic setting by 106 dermatologists with more than 5-year experience in dermoscopy recruited from 14 different European countries [10]. Each dermatologist, blinded for the histological diagnosis, was required to assess the presence or absence of 11 dermoscopic accepted to be suggestive of malignancy (Table 1), including: atypical network (ANet), irregular streaks (IS), irregular dots and globules (IDG), blue-white veil (BWV), blue-grey peppering (BGP), white scar-like areas (WSA), shiny white streaks (SWS), atypical vascular pattern (AVP), irregular blotches (IB) and regression structures (RS). During tele-testing, a total of 30 blinded cases (Fig. 1), matched with data concerning patient age, sex, maximum diameter of the lesion and body location, was randomly assigned to each participant dermoscopist.

**Table 1:** Definition of 11 dermoscopic features (adapted from ref.23, 24)

Dermoscopic feature	Abbreviation	Definition
Atypical network	ANet	Network with increased variability in the color, thickness, and spacing of the lines of the network; asymmetrically distributed; gray color
Irregular streaks	IS	Peripheral brownish to black lines/pseudopods of variable thickness and length, not combined with pigment network lines
Irregular dots and globules	IDG	Sharply circumscribed, round to oval, brown to black structures of variously sized and irregular distribution (i.e., neither distributed all over the lesion nor clustered at the center of the lesion, nor located on the network lines)
Blue-white veil	BWV	An irregular shaped blotch of blue hue with an overlying whitish ground-glass haze
Blue-grey peppering	BGP	fine dots with a blue–gray color (i.e., pepper-like structures)
White scar-like area	WSA	Irregular areas with a scar-like appearance and white to whitish color
Shiny white streaks	SWS	Short discrete white lines oriented parallel and orthogonal (perpendicular) to each other, of shiny white colour, seen only under polarized dermoscopy
Atypical vascular pattern	AVP	presence of polymorphic vessels (i.e., two or more of the following type of vessels: linear-irregular, hairpin, dotted, linear, corkscrew) and/or milky-red areas not within regression structures
Irregular blotches	IB	More than one blotch (i.e., dark structureless area) or a blotch that is located off the center
Regression structures	RS	White scar-like depigmentation and/or blue pepper-like granules usually corresponding to a clinically flat part of the lesion

### Body distribution analyses

The anatomical location was indicated for each case according to 16 anatomical sites (Table 2), including: scalp, ear, neck, shoulder, back, chest, abdomen, side, bottom, arm, forearm, back of the hand, tight, leg, ankle, back of the foot; six sites were grouped due to low size (i.e., ankle + back of the feet, scalp + ear and tight + leg). All 16 sites were then grouped into 4 anatomical areas: “upper extremities”, “lower extremities”, “posterior trunk”, “anterior trunk”.

**Table 2:** Distribution of dermoscopic structures in 268 early melanomas at 16 body sites, according to 720 evaluations (*N*) of 106 experienced dermoscopists: descriptive statistics (*n*, %)

Dermoscopic feature	Abdomen	Ankle + back of the feet	Arm + forearm	Back	Bottom	Chest	Back of the hand	Scalp + ear	Thigh + leg	Neck	Shoulder	Side
	*n* = 81	*n* = 8	*n* = 110	*n* = 226	*n* = 6	*n* = 79	*n* = 1	*n* = 14	*n* = 136	*n* = 4	*n* = 31	*n* = 21
ANet	54 (66.7%)	5 (62.5%)	84 (76.4%)	188 (83.2%)	4 (66.7%)	56 (70.9%)	1 (100%)	13 (92.9%)	101 (74.3%)	3 (75.0%)	22 (71.0%)	18 (85.7%)
IS	34 (42.0%)	4 (50.0%)	51 (46.4%)	106 (46.9%)	1 (16.7%)	31 (39.2%)	0 (0%)	8 (57.1%)	64 (47.1%)	2 (50.0%)	13 (41.9%)	7 (33.3%)
IDG	39 (48.1%)	4 (50.0%)	63 (57.3%)	121 (53.5%)	2 (33.3%)	28 (35.4%)	1 (100%)	5 (35.7%)	74 (54.4%)	1 (25.0%)	12 (38.7%)	6 (28.6%)
BWV	29 (35.8%)	1 (12.5%)	44 (40.0%)	91 (40.3%)	2 (33.3%)	27 (34.2%)	1 (100%)	5 (35.7%)	48 (35.3%)	3 (75.0%)	13 (41.9%)	5 (23.8%)
BGP	12 (14.8%)	0 (0%)	29 (26.4%)	69 (30.5%)	2 (33.3%)	8 (10.1%)	1 (100%)	2 (14.3%)	25 (18.4%)	0 (0%)	4 (12.9%)	2 (9.5%)
WSA	18 (22.2%)	1 (12.5%)	52 (47.3%)	108 (47.8%)	2 (33.3%)	18 (22.8%)	1 (100%)	3 (21.4%)	45 (33.1%)	0 (0%)	14 (45.2%)	6 (28.6%)
SWS	13 (16.0%)	0 (0.0%)	23 (20.9%)	47 (20.8%)	2 (33.3%)	16 (20.3%)	0 (0%)	2 (14.3%)	36 (26.5%)	0 (0%)	9 (29.0%)	4 (19.0%)
AVP	8 (9.9%)	0 (0.0%)	27 (24.5%)	48 (21.2%)	1 (16.7%)	8 (10.1%)	1 (100%)	1 (7.1%)	41 (30.1%)	0 (0%)	14 (45.2%)	2 (9.5%)
IB	32 (39.5%)	6 (75.0%)	54 (49.1%)	135 (59.7%)	2 (33.3%)	35 (44.3%)	1 (100%)	7 (50.0%)	80 (58.8%)	2 (50%)	11 (35.5%)	13 (61.9%)
RS	25 (30.9%)	1 (12.5%)	72 (65.5%)	139 (61.5%)	2 (33.3%)	34 (43.0%)	1 (100%)	6 (42.9%)	60 (44.1%)	3 (75%)	19 (61.3%)	8 (38.1%)

*ANet* atypical network, *IS* irregular streaks, *IDG* irregular dots and globules, *BWV* blue-white veil, *BGP* blue–grey peppering, *WSA* white scar-like areas, *SWS* shiny white streaks, *AVP* atypical vascular pattern, *IB* irregular blotches, *RS* regression structures

### Statistical analysis

Power analysis was performed with G*power. In particular, a minimum sample size of 646 was estimated, based on 5% first type error, 80% power, three degrees of freedom and a very small effect size of 0.13: this allowed us to identify small differences between % as significant. Descriptive statistics was carried out over the whole dataset. Chi-squared test was performed to evaluate the association between the features’ presence and the anatomical distribution. When association between clinical variables and body location was significant, multiple Fisher exact tests were performed to evaluate which groups were different from the other and the false discovery rate correction was performed to control the type I error [4]. To further investigate, ANOVA and chi-squared test were respectively carried out to assess the possible age and sex confounding in the evaluation of statistical differences between the four body area groups. If age and/or sex were confounding, post hoc multiple comparison with Tuckey’s procedure and/or Fisher exact tests with false discovery rate correction were respectively performed. If age/sex were confirmed as confounding factor a stratified analysis by sex/age was also carried out using the same tests of the overall analysis. Logistic regressions were performed to estimate the crude odds ratio (OR) and the adjusted odds ratio (aOR) with their 95% confidence interval (CI). The OR described the association between dermoscopic features and body group (one vs the other); after that the OR was adjusted for age and sex. A *p* value < 0.05 was considered significant. The analyses were carried out by R version 3.3.3.

## Results

The distribution of 10 dermoscopic structures in EMs located at 16 different body sites was reported in Table 2: the frequency of recognition is estimated according to 717 evaluations performed by 106 experienced dermoscopists. There was only one EM of the hand, showing all features except for IS and SWS. Excluding the head site (i.e., case number statistically not significant) the highest range of recognition was achieved by the AVP (9.9–45.2%) followed by BWV and REG (same range of 12.5–75%), and WSA (range 12–47.8%).

In Table 3 were reported the rates of recognition of ten dermoscopic features of EMs grouped per anatomical area. In general, EMs of the anterior trunk exhibited ANet in 72.3% of cases, followed by IS (41%) and IDG (40%). EMs of the rear trunk exhibited ANet in 81.3% of cases, followed by IB (56%) and IDG (50%). Concerning EMs of the upper extremities, they showed ANet in 77% of cases, followed by RS (66%) and IDG (58%). As per lower extremities, the most common features were ANet (74%), followed-by IB (60%), IDG (54%). The results of chi-square tests suggested that, when comparing anterior trunk vs rear trunk vs upper extremities vs lower extremities, six out of ten dermoscopic structures significantly differ in their distribution, i.e., IDG, BGP, WSA, RG, IB and VSA. According to the post hoc multiple comparisons, IDG, WSA and AVP showed three out of six significant paired comparisons. In particular, the distribution of AVP, IB and IDG was significantly higher on the rear trunk than on extremities and anterior trunk; RS was significantly higher on the rear trunk than on the anterior trunk/upper extremities; BGP was significantly higher on the rear trunk compared with the anterior trunk. No significant differences were found for ANet, IS and BWV according to this classification. According to the ANOVA test, age did not prove to be a confounding factor: indeed, *p* value was 0.06 and the mean age in each group was ~ 55 years. The association between sex and groups was instead significant (Chi-square test, *p* = 0.003). In particular, the “Lower extremities” group was different from all the others and it was formed by 60% female and 40% male patients; instead, the other groups had 40% women and 60% men. The post hoc multiple comparison analysis showed that “Lower extremities” was statistically different from the “Upper trunk” (*p* = 0.015). However, the stratified results should be interpreted with caution due to the decreasing power into the groups (that is about 65%).

**Table 3:** Distribution of dermoscopic structures in 268 early melanomas at 4 body areas, according to the dermoscopic evaluations (*N*) of 106 experienced dermoscopists: descriptive statistics (*n*, %), Pearson X-square test (*p*) and post hoc multiple comparison (^c,e,f^)

Body area	Upper extremities	Lower extremities	Rear trunk	Anterior trunk	*p*
Body sites	Arm, forearm, back of the hand	Thigh, leg, ankle, back of the feet	Neck, ear, shoulder, back, bottom	Chest, side, abdomen, scalp	
No. observation	*n* = 111	*n* = 144	*n* = 267	*n* = 195	
1. ANet	85 (76.6%)	106 (73.6%)	217 (81.3%)	141 (72.3%)	0.113
2. IS	51 (45.9%)	68 (47.2%)	122 (45.7%)	80 (41.0%)	0.657
3. IDG	64 (57.7%)	78 (54.2%)	136 (50.9%)	78 (40.0%)	0.009^c,e,f^
4. BWV	45 (40.5%)	49 (34.0%)	109 (40.8%)	66 (33.8%)	0.315
5. BGP	30 (27.0%)	25 (17.4%)	75 (28.1%)	24 (12.3%)	0.000^f^
6. WSA	53 (47.7%)	46 (31.9%)	124 (46.4%)	45 (23.1%)	0.000^c,e,f^
7. SWS	23 (20.7%)	36 (25.0%)	58 (21.7%)	35 (17.9%)	0.469
8. AVP	28 (25.2%)	41 (28.5%)	63 (23.6%)	19 (9.7%)	0.000^c,e,f^
9. IB	55 (49.5%)	86 (59.7%)	150 (56.2%)	87 (44.6%)	0.021
10. RS	73 (65.8%)	61 (42.4%)	163 (61.0%)	73 (37.4%)	0.000^c,f^

Multiple post hoc comparisons: upper extremities vs anterior trunk (c), lower extremities versus anterior trunk (e), rear vs anterior trunk (f)

*ANet* atypical network, *IS* irregular streaks, *IDG* irregular dots and globules, *BWV* blue–white veil, BG*P* blue-grey peppering, *WSA* white scar-like areas,* SWS* shiny white streaks, *AVP* atypical vascular pattern, *IB* irregular blotches, *RS* regression structures

The results of the association analysis between the ten dermoscopic features and the four anatomical distribution were reported in Table 4: crude odds ratios (OR) were calculated, along with adjusted odds ratio for sex and age (aOR). In the “upper extremities” area, the WSA feature was significantly prevalent compared with the other three body areas, according to OR and aOR; IS and IDG prevalence increased significantly with age (aOR). In the “lower extremities” area, the AVP feature was significantly prevalent, while the RS feature was significantly less represented than the other (OR 0.63; 95% CI 0.43–0.91) IS and IDG ORs were adjusted by age (aOR) while IB by sex (women had a major prevalence of IB) and age. In the “posterior trunk”, the features related to regression phenomenon such as RS, BGP and WSA were significantly prevalent according to both OR and aOR; moreover, IS and IDG ORs were adjusted by age (aOR) while IB by sex (women had a major prevalence of IB) and age. Finally, EMs in the “anterior trunk” area overall exhibited low rates of recognition of dermoscopic features: in particular, 6/10 features (IDG, BGP, WSA, AVP, IB, RS) were significantly less represented in the anterior trunk compared with other body areas.

**Table 4:** Analysis of association between 717 observations of dermoscopic features and different body sites, grouped per anatomic body area: crude odds ratio (OR), adjusted odds ratio for sex and age (aOR) and 95% confidence interval (CI) are calculated for each body group versus all the other 3 groups

Body area	Upper extremities		Lower extremities		Posterior trunk		Anterior trunk	
Body sites	Arm, forearm, back of the hand		Thigh, leg, ankle, back of the feet		Neck, ear, shoulder, back, bottom		Chest, side, abdomen, scalp	
	OR (IC 95%)	aOR (IC 95%)	OR (IC 95%)	aOR (IC 95%)	OR (IC 95%)	aOR (IC 95%)	OR (IC 95%)	aOR (IC 95%)
1. ANet	1.00 (0.62–1.61)	1.01 (0.63–1.64)	0.82 (0.54–1.25)	0.82 (0.54–1.24)	**1.54 **(1.06–2.24)	**1.55 **(1.07–2.26)	0.73 (0.50–1.06)	0.72 (0.49–1.05)
2. IS	1.06 (0.71–1.59)	1.05 (0.70–1.57)	1.13 (0.79–1.63)	1.12 (0.78–1.62)	1.06 (0.78–1.44)	1.07 (0.78–1.45)	0.81 (0.58–1.13)	0.82 (0.59–1.15)
3. IDG	1.46 (0.97–2.20)	1.47 (0.98–2.22)	1.25 (0.87–1.81)	1.24 (0.86–1.79)	1.09 (0.80–1.47)	1.10 (0.81–1.49)	0.59 (0.42–0.82)	0.58 (0.42–0.82)
4. BWV	1.16 (0.77–1.76)	1.18 (0.78–1.78)	0.83 (0.56–1.22)	0.83 (0.57–1.22)	1.25 (0.92–1.71)	1.25 (0.92–1.71)	0.80 (0.57–1.14)	0.79 (0.56–1.12)
5. BGP	1.44 (0.91–2.29)	1.46 (0.92–2.32)	0.72 (0.45–1.16)	0.72 (0.45–1.15)	**1.83 **(1.23–2.63)	**1.85 **(1.29–2.66)	0.42 (0.26–0.68)	0.42 (0.26–0.67)
6. WSA	**1.66 **(1.11–2.50)	**1.66 **(1.11–2.50)	0.74 (0.50–1.10)	0.74 (0.50–1.09)	**1.84 **(1.35–2.52)	**1.85 **(1.35–2.52)	0.40 (0.28–0.59)	0.40 (0.28–0.59)
7. SWS	0.97 (0.59–1.59)	0.96 (0.58–1.58)	1.31 (0.86–2.02)	1.33 (0.87–2.05)	1.05 (0.73–1.52)	1.04 (0.72–1.51)	0.76 (0.50–1.15)	0.76 (0.50–1.16)
8. AVP	1.33 (0.83–2.12)	1.33 (0.83–2.13)	**1.68 **(1.10–2.54)	1.66 (1.09–2.52)	1.27 (0.88–1.83)	1.28 (0.89–1.85)	0.32 (0.19–0.53)	0.32 (0.19–0.53)
9. IB	0.86 (0.57–1.29)	0.86 (0.57–1.30)	1.43 (0.99–2.07)	1.40 (0.97–2.04)	1.25 (0.92–1.69)	1.27 (0.93–1.72)	0.64 (0.46–0.89)	0.64 (0.46–0.89)
10. RS	2.0 (1.31–3.05)	2.0 (1.31–3.05)	0.63 (0.43–0.91)	0.63 (0.43–0.91)	**1.84 **(1.35–2.50)	1.84 (1.35–2.50)	0.45 (0.32–0.64)	0.45 (0.32–0.64)

*ANet* atypical network, *IS* irregular streaks, *IDG* irregular dots and globules, *BWV* blue-white veil, *BGP* blue–grey peppering, *WSA* white scar-like areas, *SWS* shiny white streaks, *AVP* atypical vascular pattern, *IB* irregular blotches, *RS* regression structures

## Discussion

It is generally recognized that MM location depends on environmental, genetic, social and demographic factors. Among these, the sun-exposure factor is supposed to have a non-linear relation with MM development, but rather a S-shaped curve: indeed, intermittent sun exposure seems to correlate with higher MM incidence before the age of 50, while in the elderly it is slightly prevalent on continuously exposed site, suggesting that an intermittently exposed body site can achieve the same pathogenetic doses as usually exposed skin [2, 8, 10, 30].

The analyses of distribution and association of ten dermoscopic features conventionally suggestive for malignancy, here carried out on a representative sample of de novo EMs, revealed several interesting findings.

First, the distribution analysis of dermoscopic features according to 16 body sites (Table 2) revealed that:

- EMs of the extremities exhibited the overall higher % of dermoscopic features, compared with anterior trunk which appeared globally “featureless”;
- Four out of ten dermoscopic features varied among body sites, specifically AVP, BWV, RS and WSA; AVP achieved the highest % on the shoulder and the lowest % on the abdomen [15]; BWV and RS had the lowest % on the ankle/back of the feet and the highest % on the neck [1]; WSA presence was highest on the back and lowest on the ankle/back of the feet;
- ANet, IS and SWS features were homogeneously distributed within the EMs occurred at different sites (i.e., differential presence range < 30%).

Second, the distribution analysis of dermoscopic features according to four anatomic body areas (Table 3), showed that:

- The posterior trunk had the highest frequencies of recognition of dermoscopic features related to regression and chronic traumatism (i.e., BGP, RS, WSA); [1, 15, 25, 29]
- The upper extremities showed a prevalence of early atypical lentiginous features (i.e., ANet, IS, IDG and IB); the AVP feature is significantly more prevalent in the extremities compared with the trunk;
- All dermoscopic structures were overall poorly displayed by EMs of the anterior trunk.

Third, the association analysis of dermoscopic features according to four anatomical body areas adjusted per age and sex (Table 4) confirmed that:

- The IDG feature increased with age in all body areas;
- The IB feature was prevalent on the lower legs of females and increased with age;
- The regression features (i.e., BGP, RS, WSA) were significantly prevalent in the posterior trunk than in the three other groups, independently from age and sex;
- The anterior trunk was “featureless” for six out of ten dermoscopic features.

Taken together, the present findings could be, at least partially, interpreted according to the hypothesis of a non-linear but S-shaped correlation between MM body distribution and UV-cumulative rates [3, 7, 8], that could explain: the higher rates of lentiginous-related features—ANet and IS on the chronically exposed upper extremities, and the predominance of IDG and IB at lower extremities and posterior trunk, intermittently exposed. In particular, the IB feature can be regarded as indicative of EM when detected in the lower female leg with advanced age, while the IDG feature increased with age independently from the body location, as a superficial-growing-related feature. In addition, MM of the anterior trunk seem to be featureless at early stages according to the dermoscopic features conventionally assumed as suggestive of malignancy. Thus, it could be questioned if the current pattern analysis could not sufficiently and accurately detect EMs arising on the anterior trunk, especially photo-protected areas such as the abdomen and the side.

This study had several limitations. First, although the number of EMs lesions selected was enough to obtain a power of 80%, the power decreased to 65%. Stratifying into male and female subgroups. Some body sites (head, neck, ankle/back of the feet) had a lower number of cases compared with the other more represent sites (i.e., back, legs): however, we preferred not to normalize all 16 body sites groups with the same number of lesions, but to maintain the collection rate to respect the distribution of EMs encountered in clinical practice. It should be also underlined that dermoscopists were blinded for the histological diagnosis but were aware of the exact body site, also having the clinical picture of the body area available in the tele-dermoscopic test: it may be questioned if this fact could somehow affect the dermoscopic assessment but was specifically designed to reproduce the daily activity of dermatologists in clinical practice.

## Conclusions

The body location has an impact on the dermoscopic appearance of EM. Globally, EMs on the extremities show a variable prevalence of the dermoscopic features that are conventionally referred to as indicative of malignancy. These are which are difficult to observe in EMs arising on the abdomen, chest and side in the early stages. The EMs on the lower leg of women showed an age-related increase of the irregular blotches feature. Further studies focused on a broader data set of trunk EMs may be needed to provide new dermoscopic features that can detect malignant changes in the early stages.

## Declarations

### Research involving human and animal participants

All procedures performed in this study involving human participants were in accordance with the local ethical committee and with the 1964 Helsinki Declaration and its later amendments or comparable ethical standards.
